# Supplementary material for: Anterior Temporal Lobe Morphometry Predicts Categorization Ability
Source: Front Hum Neurosci. 2018 Feb 7;12:36. doi: 10.3389/fnhum.2018.00036 (PMC5808329; doi:10.3389/fnhum.2018.00036)
Supplement: Supplementary file 1 [file Data_Sheet_1.docx]

**SUPPLEMENTARY MATERIAL**

1. **Supplementary method.**

| **taxonomic categories** | | **thematic categories** |
| --- | --- | --- |
| animals | insects | found on the beach (crab and shell) |
| athletes (gymnasts and joggers) | jewelleries | in relation to aeronautics (space rockets and plane) |
| balls | jungle mammals | in relation to baseball |
| birds | lamps | in relation to breakfast |
| body parts | leather goods | in relation to brushing tooth |
| carafes | liquid container | In relation to cloakroom (hanger and coat hack) |
| childrens' games | mammals | in relation to closet |
| clothes | men accessories (tie and bow tie) | in relation to clothes |
| clothing accessories | molluscs | in relation to Egypt |
| dinosaurs | money (coin and banknote) | In relation to Egypt (pyramid and mummy) |
| dishes | music instruments | in relation to face (nose and eye) |
| dogs | niches for animals | in relation to feet (shoes/socks) |
| domestic animals | office equipment | in relation to fishing |
| doorhandles | optic (microscope and magnifying glass) | in relation to flame (candle/matchstick) |
| dry fruits | plants | in relation to ice-cream |
| electrical households | plasters | in relation to ironing |
| evening clothes | produces | in relation to japan |
| exotic fruits | pyramids | in relation to legends (unicorn and pegasus) |
| famous monuments | religious buildings | in relation to light (bulb and oil lamp) |
| farm animals | reptiles | in relation to locks |
| feline | rodents | in relation to mail (stamp and envelop) |
| fishes | sea animals (fishes and dolphin) | in relation to money |
| flowers | sea mammals | in relation to mythology |
| food | signals | in relation to painting |
| frogs | storage units | in relation to party/celebration |
| fruits | tables | in relation to pirates |
| furniture | tools | in relation to post |
| games | two wheels transportations | in relation to press (journal and magazin) |
| gardening tools | vegetables | in relation to rugby |
| geishas | vegetables/mushrooms | in relation to sewing (thread/thimble) |
| hats | weapons | in relation to sherif (hat and star) |
| ice creams | women's clothes | in relation to sink (sink and tap) |
|  |  | in relation to space (space rocket and astronaut) |
|  |  | in relation to the beach (parasol and sunlornger) |
|  |  | in relation to transportation |
|  |  | in relation to travel |
|  |  | in relation to trees |
|  |  | in relation to wine |
|  |  | objects used to cut (cisors and utility knife) |
|  |  | in relation to head (brain and face) |
|  |  | in relation to birds (cage and nest) |
|  |  | things that are found in the sky |
|  |  | in relation to heat (fireplace and heating) |

1. **Supplementary results:**
   1. ***Correlation with age.***

To investigate the relationship between VBM regional gray matter (GM) structural variability and age more thoroughly, we ran multiple regression analyses in SPM8 between GM volume and age, with education and sex as covariates of non-interest. At an FWE-corrected threshold, we observed negative correlation between age and grey matter volume in the prefrontal cortex.

***Supplementary figure 1*. Negative correlation with age (p < 0.05 after FWE correction at the cluster level)**

**
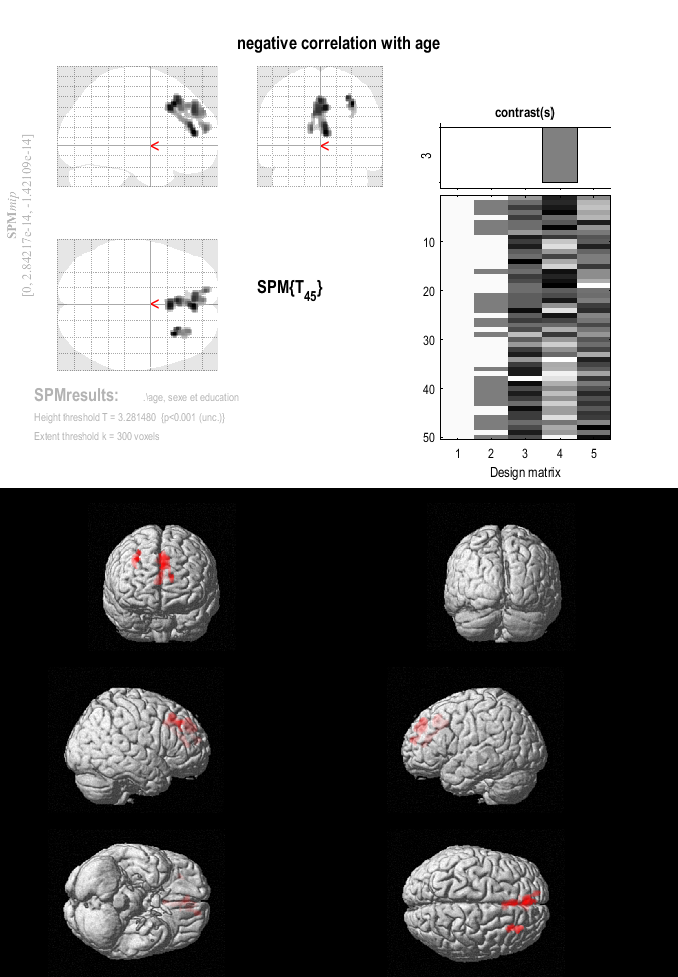
**

***Supplementary table 1*.** Negative correlation with age (p < 0.05 after FWE correction at the cluster level)

| **brain region** | **side** | **BA** | **MNI coordinate** | **T value (peak)** | **cluster size** | **cluster-level p(FWE)** |
| --- | --- | --- | --- | --- | --- | --- |
| Middle frontal gyrus | R | 46 | 32 23 42 | 4.79 | 316 | 0.021 |
| Anterior cingulate cortex | R | 32 | 3 44 12 | 4.76 | 823 | 0.001 |
| Superior medial frontal gyrus | L | 9 | - 3 45 36 | 4.41 | 706 | 0.021 |

- 1. ***Supplementary table 2*.** VBM whole-Brain Analysis: GM correlations with RT in *Shape* and *Category* dimensions*,* and *Same*, and *Different* conditions: additional clusters at p<0.001 uncorrected.

| ***positive correlation*** | **brain region** | **side** | **BA** | **MNI coordinate** | **T value (peak)** | **cluster size** | **cluster-level p(FWE)** |
| --- | --- | --- | --- | --- | --- | --- | --- |
| Shape | inferior frontal gyrus | R | 47 | 41 33 1 | 4.15 | 117 | ns |
| Category | precuneus | R | 5/7 | 15 -51 51 | 4.87 | 167 | ns |
|  | Precentral gyrus | R | 6 | 54 5 40 | 4.27 | 110 | ns |
|  | Inferior and middle frontal gyrus | L | 45/46 | - 39 42 13 | 4.05 | 118 | ns |
| Same | - | - | - | - | - | - | - |
| Different | - | - | - | - | - | - | - |
| ***negative correlation*** |  |  |  |  |  |  |  |
| **Shape** | **middle and inferior temporal gyrus** | **R** | **20/21** | **56 -19 -20** | **4.74** | **679** | **0.044** |
| **Category** | **Temporal pole, middle and inferior temporal gyrus** | **R** | **20/21/38** | **57 -2 -27** | **4.97** | **1558** | **0.001** |
|  | parahippocampal gyrus | R | 35/36 | 23 -19 -29 | 4.41 | 214 | ns |
|  | Hippocampus, parahippocampal gyrus and amygdala | L | 28/35/36 | -18 -3 -24 | 3.67 | 290 | ns |
| **Same** | **temporal pole, middle and inferior temporal gyrus** | **R** | **20/21** | **57 -13 -20** | **5.00** | **1352** | **0.002** |
|  | parahippocampal gyrus | R | 30/36 | 23 -19 -29 | 4.33 | 194 | ns |
|  | middle temporal gyrus | L | 21/22 | -63 -24 -3 | 4.06 | 150 | ns |
|  | Hippocampus, parahippocampal gyrus and amygdala | L | 28/35/36 | -21 -1 -27 | 3.80 | 364 | ns |
| **Different** | **middle and inferior temporal gyrus** | **R** | **20/21** | **57 -16 -21** | **4.97** | **1308** | **0.003** |
|  | parahippocampal gyrus | R | 30/36 | 21 -19 -29 | 4.04 | 114 | ns |
|  |  |  |  |  |  |  |  |

- 1. ***Supplementary table 3.*** WM correlations with RT in Shape, Category, Same, and Different tasks. Additional clusters at p < 0.001 uncorrected.

| ***positive correlation*** | **brain region** | **side** | **MNI coordinate** | **T-value (peak)** | **cluster size** | **cluster-level p(FWE)** |
| --- | --- | --- | --- | --- | --- | --- |
| **-** | - | - | - | - | - | - |
| ***negative correlation*** |  |  |  |  |  |  |
| Shape | **-** | **-** | **-** | **-** | **-** | **-** |
| **Category** | **Temporal lobe** | **R** | **48 -9 -27** | **4.92** | **689** | **0.037** |
| Same | temporal lobe | R | 51 -10 -23 | 4.40 | 385 | ns |
| Different | temporal lobe | R | 48 -11 -29 | 4.17 | 306 | ns |
|  | frontal lobe | L | -12 45 21 | 4.06 | 114 | ns |

- 1. ***Whole brain analyses at lower threshold:*** additional clusters as compared to FWE-corrected results described in the article.
     1. GM correlation with reaction time (RT) in Shape and Category dimensions (supplementary Table 1)

At p<0.001 uncorrected for multiple comparisons, minimum size 100 voxels, additional *positive correlations* were found with RTs in *Category* dimension within the right precuneus (BA 5/7), right precentral gyrus (BA 6) and left inferior and middle frontal gyri (BA 45/46). Additional positive correlations were found with RTs in *Shape* dimension within the left inferior frontal gyrus (BA47). Additional *negative correlations* were found between RTs in *Category* dimension with the left hippocampus, left amygdala and bilateral parahippocampal gyri (table 1).

- - 1. GM correlation with reaction time (RT) in *Same* and *Different* conditions (supplementary Table 1)

At p<0.001 uncorrected for multiple comparisons, minimum cluster size 100 voxels, *No positive correlation* was found with RTs in *Same* and *Different tasks*. Additional *negative correlation* were found between RTs in *Same* condition and the right parahippocampal gyrus, left parahippocampal and middle temporal gyri, left hippocampus, and amygdala, while RTs in *Different* tasks correlated negatively with the right parahippocampal gyrus.

- - 1. WM correlation with RT in *Shape, Category, Same* and *Different* tasks (supplementary table 2)

At p<0.001 uncorrected for multiple comparisons, minimum cluster size 100 voxels, no *positive correlation* was found with RTs in *Category* or *Shape* dimensions and *Same* or *Different* conditions.

Additional *negative correlation* was found with RTs in *Same* tasks and WM volume in the right temporal lobe. Additional *negative correlation* was found with RTs in *Different* tasks and WM volume in the right temporal lobe and the left frontal lobe.

1. **Supplementary figure 2. Results from the whole-brain GM VBM analysis according to dimension. P<0.05 after FWE correction.**

**
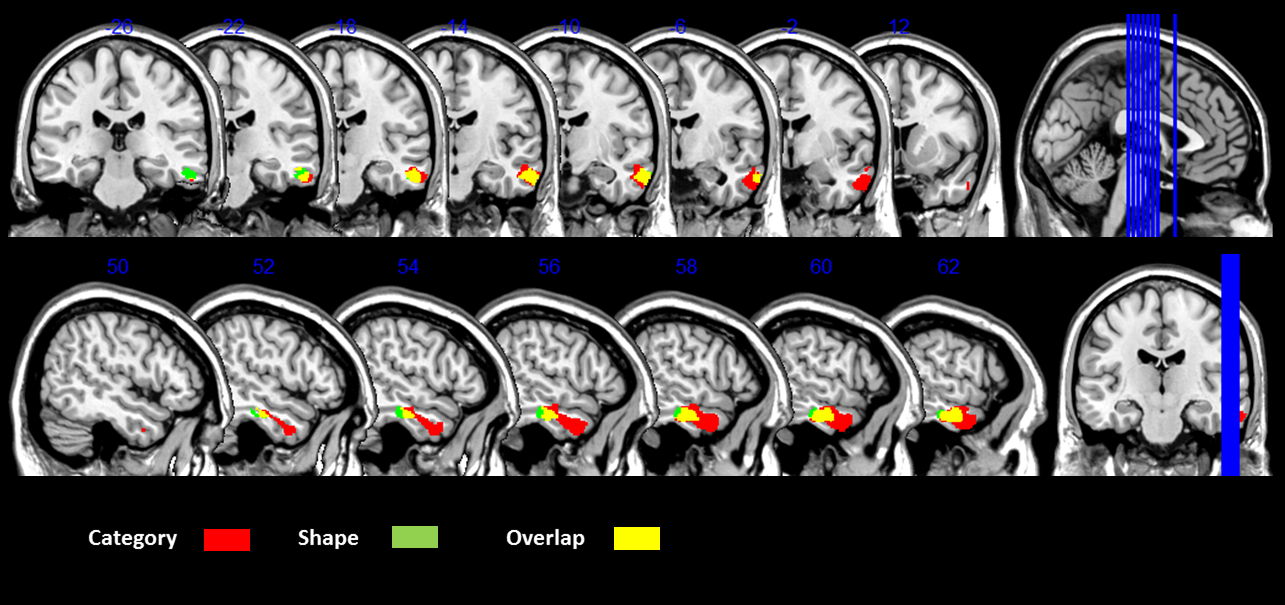
**

This figure shows additional slices compared to figure 3. Significant regions associated with changes in GM volume related to performance in terms of RT are superimposed on coronal and sagittal slices.

1. **Supplementary figure 3. Results from the whole-brain GM VBM analysis according to condition. P<0.05 after FWE correction.**


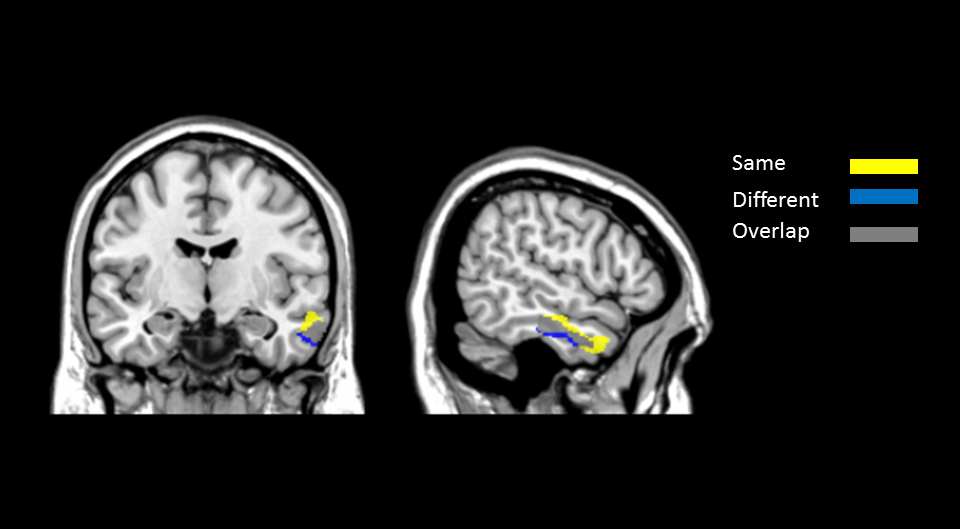


Significant regions associated with changes in GM volume related to performance in terms of RT are superimposed on a coronal (left) and sagittal (right) view.

GM volume was negatively correlated with RT in *Same* (yellow) and *Different* (blue) conditions. Shared regions are shown in gray.
